# MOD-LSP, MODIS-based parameters for hydrologic modeling of North American land cover change

**DOI:** 10.1038/s41597-019-0150-2

**Published:** 2019-08-09

**Authors:** Theodore J. Bohn, Enrique R. Vivoni

**Affiliations:** 1 School of Earth and Space Exploration, Arizona State University, Tempe, AZ 85287 USA; 2 School of Sustainable Engineering and the Built Environment, Arizona State University, Tempe, AZ 85287 USA

**Keywords:** Hydrology, Environmental impact, Hydrology, Phenology, Biogeography

## Abstract

Earth systems models require gridded land surface properties to compute fluxes of water, energy, and carbon within the landscape and to the atmosphere. However, most parameter sets contain time-invariant properties despite their known variability. Here we present new MODerate Resolution Imaging Spectroradiometer (MODIS)-based land surface parameters (MOD-LSP) formatted for the Variable Infiltration Capacity (VIC) hydrologic model that account for seasonal and interannual variability and longer-term change over the continental United States, Mexico, and southern Canada at 0.0625° spatial resolution and monthly temporal resolution. MOD-LSP improves over previously-available parameter sets via: (1) land cover maps of higher native spatial resolution; (2) multiple versions corresponding to the land cover of years 1992, 2001, and 2011; (3) spatially-explicit mean annual cycles of land surface properties, including leaf area index, canopy fraction, and albedo, derived from 17 years of observations; and (4) additional 17-year time series of these properties. The MOD-LSP parameters are useful as inputs to the VIC model, as an example land surface scheme, to assess the hydrologic impacts of land cover change from interannual to decadal scales; and as stand-alone datasets characterizing the temporal variability of these properties as a function of land cover class.

## Abstract (summary)

**Table:** 

Design Type(s)	data integration objective • modeling and simulation objective • statistical parameter determination objective
Measurement Type(s)	Parameter
Technology Type(s)	computational modeling technique
Factor Type(s)	
Sample Characteristic(s)	North America • land

Machine-accessible metadata file describing the reported data (ISA-Tab format)

## Background & Summary

Hydrologic and earth system models require specification of gridded land surface properties such as land cover type, leaf area index (LAI), and albedo to simulate the land surface response to meteorological forcings over watershed to global scales, typically at resolutions of less than 10 km. For those properties that can be measured from space, remote sensing products have been useful in providing gridded values at high resolution with global coverage^1–7^. However, most land surface parameters represent a “snapshot” from a specific time period that remains static in multi-decadal simulations, despite their known temporal variability^8^. Furthermore, for studies at regional to global scales, the effort required to process the large data volume of remote sensing datasets can discourage the updating of these parameters, in some cases leading to the use of land surface parameters that represent time periods outside of the period of simulation.

One such land surface model is the Variable Infiltration Capacity (VIC) model^9,10^. Prior VIC parameters over the United States^11–13^, Mexico^14^, and North America^15^ share several limitations: (1) they were derived from a coarse-resolution land cover map, based on Advanced Very High-Resolution Radiometer (AVHRR) imagery from the early 1990s^7^; (2) the annual cycle of monthly LAI values was derived by spatially interpolating a sparse (<100 points over North America) subset of observations from a single year of an AVHRR-based dataset^16^; (3) in all land cover classes (except bare soil), the vegetation canopy was assumed to have 100% areal coverage; (4) each land cover class was assigned a single spatially (and temporally, for most classes) invariant albedo obtained from literature values; and (5) the urban land cover class was replaced by bare soil.

In addition, the prior efforts have not accounted for land cover variability and change, including: (1) land cover conversions in response to natural or human disturbance; and (2) interannual variations in phenology (seasonally-varying plant characteristics such as LAI, canopy fraction, and albedo) in response to climate fluctuations. Several efforts in the remote sensing community have provided sequential land cover classifications over multiple decades using consistent methodology. For instance, the National Land Cover Database (NLCD)^2^ contains consistent classifications over the United States for the years 1992, 2001, 2006, 2011, and 2016; while the Instituto Nacional de Estadística y Geografía (INEGI) Uso del Suelo y Vegetación land cover product^17^ contains consistent classifications over Mexico for years 1985, 1993, 2002, 2007, and 2011. Similarly, interannual variability in phenology from the MODerate Resolution Imaging Spectroradiometer (MODIS) has received attention in regional hydrologic modeling studies^18–26^.

Here, we describe new MODIS-based land surface parameters for the VIC model (MOD-LSP), covering the continental United States, Mexico, and southern Canada at 0.0625° (6 km) resolution. MOD-LSP is formatted to be compatible with the image driver of VIC model release 5.0 and later^10^. Its spatial domain and grid resolution are compatible with gridded daily meteorological forcings and hourly disaggregation parameters over North America^15,27,28^. MOD-LSP overcomes the limitations of prior parameter sets and facilitate studies of land cover variability and change. To do so, several versions of the MOD-LSP parameters based on NLCD and INEGI classifications from years 1992/3, 2001/2, and 2011^2,17^ are available, as well as a land cover classification based on MODIS^1^. In terms of phenology (LAI, canopy fraction, and albedo), MOD-LSP contains the following improvements: (1) canopy fraction (*f*_*canopy*_) and albedo, which were not derived from remote sensing in prior studies, are now included; (2) the long-term mean annual cycles of phenology variables have been derived from 17 years (2000–2016) of 8- and 16-day MODIS products using all available pixels, yielding spatially explicit and statistically representative estimates for each land cover class in each grid cell; (3) phenology values have been provided for urban areas; and (4) to account for interannual variability in phenology, monthly time series of phenology spanning the period 2000–2016 have been generated as an optional set of vegetative forcings for the VIC model.

## Methods

**Fig. 1:**
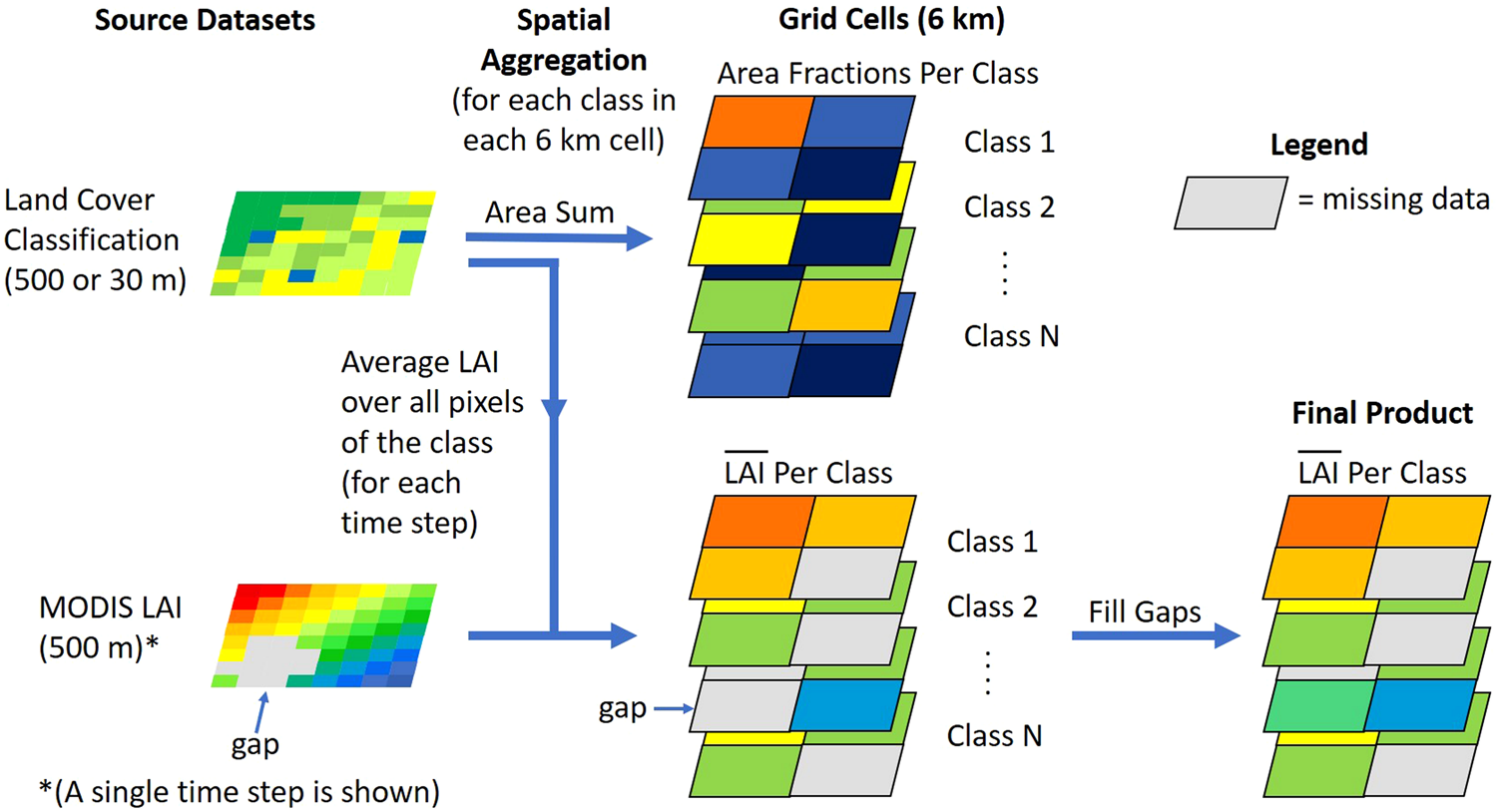
Example process flow for LAI, showing spatial aggregation of MODIS LAI over a land cover classification to generate spatial average values for each class at 6 km resolution (only a single time step of MODIS LAI is shown).

Several spatial datasets were used in creating and evaluating the parameters at 0.0625° (6 km) resolution. Details of satellite sensors, spatial resolution, and acquisition dates for these datasets can be found in Table 1. Processing of the land surface properties centered around spatial aggregation, i.e., grouping the 500 m MODIS observations by the classes given by the 30 or 500 m land cover pixels within each 6 km grid cell, yielding a separate spatial average for each class within the cell (Fig. 1). Before the spatial aggregation step, the NLCD and INEGI land cover classifications were harmonized to a common legend. After aggregation, data gaps were filled via temporal and spatial interpolation, separately for each land cover class. Details of these procedures are given below.

**Table 1:** Descriptions of spatial datasets used in this study.

Type	Abbreviation	Dataset	Reference	Satellite Source	Acquisition Date Range	Temporal Interval	Resolution
Land Cover Classification	UMD	University of Maryland Land Cover	^7^	AVHRR	1981–1994	Single map	1 km
	MOD12Q1.051	MODIS Collection 5 Global Land Cover	^1^	MODIS	2001–2013	Annual	500 m
	NLCD	National Land Cover Database	^2, 31, 32^	LANDSAT	1992–2011	Years 1992, 2001, 2006, 2011	30 m
	INEGI	Uso del Suelo y Vegetación	^17^	LANDSAT	1992–2011	Years 1993, 2002, 2007, 2011	n/a (imagery delineated by hand into polygons)
Static Surface Properties	GTOPO30	Global 30 Arc-Second Digital Elevation Model	^6^	n/a	n/a	Single map	30″ (1 km)
	NLCD-Forest	National Land Cover Database USFS Tree Cover, Cartographic	^48^	LANDSAT	2008–2009	Single map	30 m
	NLCD-Shrub	National Land Cover Database Shrub Cover	^49^	WorldView-2, LANDSAT	2013	Single map	30 m
	FAO	FAO-UNESCO Digital Soil Map of the World	^35^	n/a	n/a	Single map	5′ (8 km)
Time-Varying Surface Properties	B2002	Leaf Area Index	^16, 34^	AVHRR	1981–1994	10 day	5′ (8 km)
	MCD15A2H.006	Leaf Area Index	^3^	MODIS	2000–2016	8 day	500 m
	MOD13A1.006	Normalized Difference Vegetation Index	^4^	MODIS	2000–2016	16 day	500 m
	MCD43A3.006	Albedo	^5^	MODIS	2000–2016	1 day	500 m

### Spatial domain

**Fig. 2:**
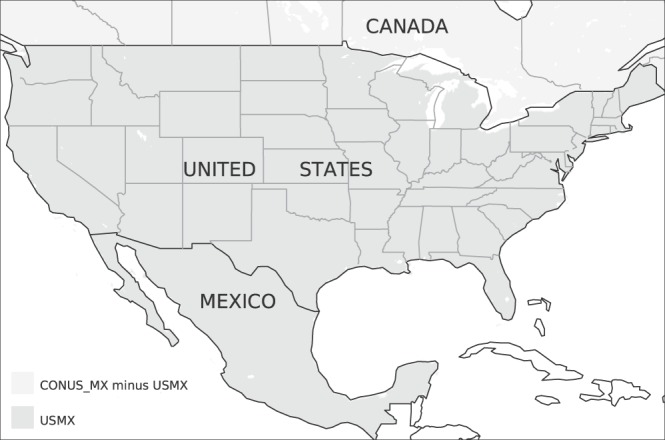
Spatial extents of the USMX (dark gray region) and CONUS_MX (union of dark and light gray regions) domains.

The spatial domain spans the region bounded by 14.5° and 53°N latitude and 125° and 67°W longitude (Fig. 2). This domain is an extension of the CONUS domain, over which several gridded meteorology and hydrology datasets^11–13,29^ have been constructed, to include Mexico; hence we will refer to the domain as CONUS_MX hereafter. In those cases for which our analysis omits southern Canada, we will refer to that subset of the domain as USMX.

### VIC parameters

The VIC parameter datasets (Table 2) include “L2015”, the parameter set used in Livneh *et al*.^15^, covering the CONUS_MX domain; and “MOD-LSP”, the suite of new parameter sets with phenology based on MODIS observations, covering either CONUS_MX or USMX domains, depending on the underlying land cover classifications.

**Table 2:** Description of various versions of VIC input parameters.

Land Cover Code (*lc_source*.*lc_year*)	Domain	Soil Properties	Land Cover Classification			Phenology			Comment
			Source	Temporal Coverage	Classification Scheme	Source	Seasonal cycle	Time Series	
L2015	CONUS_MX	FAO	UMD	Single snapshot representing 1981–1994	UMD, with open water and urban replaced by bare soil	LAI: AVHRR *f*_*canopy*_: n/a Albedo: n/a	Single cycle from 1992–1993	n/a	Phenology derived by spatial interpolation of values from <50 pixels in each class
MOD_IGBP.mode	CONUS_MX	FAO	MODIS MOD12Q1.051	For each pixel, mode of class over 2001–2013	IGBP	LAI: MCD15A2H.006 *f*_*canopy*_: MOD13A1.006 Albedo: MCD43A3.006 (Taken from “White-Sky Albedo for shortwave broadband”)	Monthly climatology 2000–2016	Monthly, 2000–2016	
NLCD_INEGI.2011	USMX	FAO	NLCD over US; INEGI over Mexico	2011	NLCD 2011		2011	n/a	
NLCD_INEGI.2001	USMX	FAO		2001 over US; 2002 over Mexico	NLCD 2011		2001	n/a	
NLCD_INEGI.s2011	USMX	FAO		2011	NLCD 2011		Monthly climatology 2000–2016 from pixels with stable class between 2001 and 2011	Monthly, 2000–2016	
NLCD_INEGI.s2001	USMX	FAO		2001 over US; 2002 over Mexico	NLCD 2011			Monthly, 2000–2016	
NLCD_INEGI.s1992	USMX	FAO		1992 over US; 1993 over Mexico	NLCD 2011			n/a	Over US, NLCD 1992–2001 retrofit change product applied to 2001, preserving relative proportions of shrub and grass relative to their total and of crop and pasture relative to their total

All VIC parameters have 0.0625 degree (6 km) spatial resolution.

#### Land cover classifications

The land cover classification underlying the L2015 dataset was the AVHRR-based University of Maryland (UMD) land cover product^7^, modified for the North American Land Data Assimilation project (NLDAS)^12^ to exclude the open water, urban, and perennial ice/snow classes (UMD-NLDAS hereafter).

MOD-LSP parameter sets are named based on the underlying land cover classification, according to the convention (Table 3): *lc_source*.*lc_year*, where *lc_source* indicates the land cover source and *lc_year* indicates the year or set of years to which the classification pertains. Values of *lc_source* include: MOD_IGBP, based on the IGBP classification of the MOD12Q1.005 product^1^ over the CONUS_MX domain; and NLCD_INEGI, which covers the USMX domain and is based on the NLCD classification^2^ over the United States and the INEGI classification^17^ over Mexico. To combine the NLCD and INEGI products into a single product, the more numerous INEGI land cover classes were mapped to the 16 NLCD 2011 classes (e.g., all shrub classes were mapped to shrublands, etc.). Details of the procedure can be found in the NLCD_INEGI GitHub archive (https://github.com/tbohn/NLCD_INEGI/releases/tag/v1.6)^30^.

**Table 3:** Dataset file description.

Name	Description
global_param.template	Template for global parameter file for VIC model
params.*domain*.*lc_source*.*lc_year*.*phen_years*.nc	VIC soil and vegetation parameters. In these files, phenology data (LAI, *f*_*canopy*_, albedo) for each class in each grid cell consist of a single set of 12 climatological monthly mean values. File naming convention is as follows: • *domain* = domain name (“CONUS_MX” or “USMX”) • *lc_source* = land cover source name (“L2015”, “MOD_IGBP”, or “NLCD_INEGI”) • *lc_year* = code describing the year(s) of acquisition from which pixels are assigned classes; can be one of (“mode”, *year*, or “s”*year*) where: ○ *mode* = class is the most common class from all maps over the period 2000–2013 ○ *year* = class comes from the land cover map for year *year* ○ “s”*year* = same as *year*, but the “s” signifies that the phenology data were only aggregated over pixels for which land cover class was stable (did not change) between 2001 and 2011. Pixels for which land cover class changed were assigned phenology values via spatial interpolation from stable neighbors of the class to which the pixel belonged in the given *lc_year*. • *phen_years* = years of MODIS products from which phenology values are derived, in the format *startyear*_*endyear*.
veg_hist.*domain*.*lc_source*.*lc_year*.*phen_years*.monthly.tgz	Gzipped tar file of directory containing yearly files (one file per year) of time-varying monthly phenology. Individual files follow the naming convention: veg_hist.*domain*.*lc_source*.*lc_year*.*phen_years*.monthly.*year*.nc where • *domain*.*lc_source*.*lc_year*.*phen_years* = same naming convention as for the params files • *year* = year of phenology to which this file pertains Note: these files must be converted from monthly values to sub-daily values (same time step as the meteorological forcings) via the following scripts, available at https://github.com/tbohn/VIC_Landcover_MODIS_NLCD_INEGI/releases/tag/v1.6: • disagg_veghist_monthly2hourly_nc.py • wrap_disagg_veghist_monthly2hourly_nc.pl

**Table 4:** Statistics of L2015 and NLCD_INEGI.2011 mean annual and seasonal *f*_*canopy*_ values over the period 2000–2016 relative to NLCD-Shrub and NLCD-Forest products.

Land Cover Category	Parameter Set	Season	Statistics			
			μ	μ/μ_ref_	Pearson r	RMSE
Shrub	L2015	Annual	1.000	7.2368	0.0000	0.8658
	NLCD_INEGI.2011	Annual	0.0962	0.6964	0.3643	0.0940
		Winter	0.0599	0.4332	0.2591	0.1142
		Spring	0.1447	1.0472	0.3107	0.1080
		Summer	0.1116	0.8073	0.3528	0.1008
		Fall	0.0688	0.4978	0.3651	0.1063
Forest	L2015	Annual	1.000	2.2810	0.0000	0.6120
	NLCD_INEGI.2011	Annual	0.5458	1.2449	0.6893	0.2064
		Winter	0.3163	0.7214	0.3786	0.2687
		Spring	0.6560	1.4962	0.6502	0.2923
		Summer	0.7679	1.7288	0.6692	0.3772
		Fall	0.4529	1.0331	0.5877	0.1999

The subscript “ref” refers to NLCD-Shrub and NLCD-Forest. Seasonal mean values for L2015 are identical to the annual mean values.

Because the MOD12Q1.005 product provided 13 separate annual maps over 2001–2013, during which many pixels changed class multiple times, a single map representative of the entire period was created for the MOD_IGBP parameter set by assigning to each pixel the class it most frequently had over the period. This has been denoted by setting *lc_year* to “mode”. The NLCD_INEGI datasets used the NLCD maps from 1992, 2001, and 2011^2,31,32^ over the US and the INEGI maps from 1993, 2002, and 2011^17^ over Mexico. It should be noted that although both the NLCD and INEGI products were derived from Landsat imagery, they were created with different methods: NLCD was classified automatically on a pixel-by-pixel basis, while INEGI products were delineated manually into polygons. Furthermore, the original NLCD 1992 product used a different method from that used in subsequent years. For consistency with years 2001 and 2011, we generated our 1992 map by applying the NLCD “1992–2001 Land Cover Change Retrofit” product^32^ to the 2001 map. For the NLCD_INEGI parameter sets, *lc_year* was set to the year of the NLCD product used, with the INEGI products from 1993 and 2002 used in *lc_years* 1992 and 2001. Details of the procedure can be found in the NLCD_INEGI GitHub archive (https://github.com/tbohn/NLCD_INEGI/releases/tag/v1.6)^30^.

#### Time-varying surface properties

VIC requires specification of a repeating annual cycle of 12 monthly values of time-varying surface properties for each land cover class of each grid cell: LAI, albedo, roughness length, and displacement height. An additional annual cycle of canopy fraction (*f*_*canopy*_) is optional^24^, and was included in the MOD-LSP parameter sets. For roughness length and displacement height, all VIC parameter datasets used a spatially-uniform repeating annual cycle of monthly values for each land cover class, taken from literature^33^.

The L2015 parameter set contained a unique annual cycle of monthly LAI values for each land cover class in each 6 km grid cell, derived from an AVHRR-based product^34^. This annual cycle was derived from only a single year (1992–1993), and from a sparse (<50) set of pixels in each land cover class, which were extended over the CONUS_MX domain by spatial interpolation. For albedo, L2015 used a constant, spatially-uniform value for each land cover class, taken from literature^33^. L2015 did not contain values of *f*_*canopy*_, such that VIC used a default value of 1.0 everywhere.

The MOD-LSP parameter sets were based on monthly phenology from MODIS products: 8-day LAI from the MOD15A2H.006 product for the period between 2000-02-18 and 2001-06-26 and the MCD15A2H.006 product for the period 2001-07-04 through the end of 2016^3^; albedo from the “White-Sky Albedo from shortwave broadband” variable in the 1-day MCD43A3.006 product^5^ for 2000–2016; and *f*_*canopy*_ derived from Normalized Difference Vegetation Index (NDVI) of the 16-day MOD13A1.006 product^4^ for 2000–2016. For consistency with LAI, only those albedo observations corresponding to the 8-day LAI schedule were used. For those 8-day intervals when the 16-day NDVI data were unavailable, NDVI values were treated as missing data.

Phenology variables were aggregated from 500 m to the 6 km grid by computing a separate spatial average value for each land cover class in each 6 km grid cell:

1 $$\bar{x}\left(c,t,i,j\right)=\frac{1}{{N}_{l}(c)}\mathop{\sum }\limits_{{k}_{l}=1}^{{N}_{l}(c)}\,x\left({k}_{p}({k}_{l}),t\right),$$

where *x* is a phenology variable, *k*_*l*_ is an index of the set of *N*_*l*_(*c*) land cover pixels of class *c* within the 6 km cell, *k*_*p*_(*k*_*l*_) is the index within the cell of the MODIS pixel containing land cover pixel *k*_*l*_, *t* is the time index (at 8-day intervals), and *i* and *j* are the row and column indices of the grid cell. For the MOD_IGBP dataset, land cover pixels were defined on the same 500 m sinusoidal grid as phenology pixels, so that each phenology pixel corresponded to exactly one land cover pixel. For the NLCD_INEGI datasets, the smaller size of land cover pixels (30 m) led to multiple land cover pixels of potentially many different classes corresponding to a single phenology pixel and taking on the same value of *x*. This may cause some contamination of phenology values by other classes in regions where classes occur in patches smaller than the 500 m MODIS resolution.

Pixels that were contaminated by poor retrievals, clouds, or snow, as indicated by the FparLAI_QC and FparExtra_QC variables included in the MCD15A2H.006 product, were excluded from the spatial average. In some cases, this led to entire grid cells missing data for one or more dates, particularly for cells north of 50°N in winter (Supplementary Fig. 1). These missing values were filled by interpolation. Even after filtering out pixels that were flagged for snow, observations immediately before and after the flagged observations often exhibited albedo values much larger than the typical range of snow-free variability, indicating at least partial snow coverage. Therefore, after the aggregation step, we removed all data for which the albedo exceeded four standard deviations above the long-term mean (this was the smallest threshold that avoided throwing away substantial amounts of valid snow-free data).

The MCD15A2H.006 product did not provide estimates of LAI over pixels classified in the MOD12Q1.005 product as open water, barren, perennial snow/ice, urban, or unclassified. With the exception of urban pixels, LAI was set to 0 in these cases. For urban pixels, LAI was estimated via an empirical relation estimated by sampling pixels of shrubland, grassland, and forest classes surrounding Phoenix, Los Angeles, San Francisco, Portland, and Seattle: LAI = 8(NDVI − NDVI_min_)^2^, where NDVI_min_ = 0.1.

*f*_*canopy*_ was derived from NDVI as:

2 $${f}_{canopy}={\left[\left({\rm{NDVI}}-{{\rm{NDVI}}}_{{\rm{\min }}}\right)/\left({{\rm{NDVI}}}_{{\rm{\max }}}-{{\rm{NDVI}}}_{{\rm{\min }}}\right)\right]}^{2},$$

where NDVI_max_ = 0.8.

Gaps in the 8-day data were filled as illustrated in Supplementary Fig. 1: At each grid cell, standardized anomalies were computed, following:

3 $$x^{\prime} \left(c,y,d\right)=(x(c,y,d)-{\mu }_{x}(c,d))/{\sigma }_{x}(c,d),$$

where *x*′ = standardized anomaly, *c* = land cover class index, *y* = year, *d* = day of year, *x* = phenological variable, and *μ*_*x*_ and *σ*_*x*_ = climatological mean and standard deviation of *x* for class *c* and day of year *d*. All values of *μ*_*x*_ and *σ*_*x*_ for which fewer than 5 observations were available on a given day of the year were set to “missing”. In the anomaly time series, missing values either on the first or last time steps or for which the nearest valid data points were more than 2 intervals away were set to 0, and remaining gaps in the anomaly time series were linearly interpolated. Temporal gaps in *μ*_*x*_ and *σ*_*x*_ were filled by linear interpolation, treating the climatological cycle as periodic. Subsequently, anomalies were recombined with *μ*_*x*_ and *σ*_*x*_ to assemble the final gap-filled time series. Any remaining gaps (cells for which no observations were available at any time) were filled with spatial interpolation from values from the same land cover class in neighboring cells, using a Gaussian kernel with σ = 1 cell. Thus, the final gap-filled data are estimates of snow- and cloud-free values. In the case of albedo, a land surface model such as VIC will replace the input albedo with a simulated snow albedo when snow is present.

The requisite repeating annual cycle of monthly phenology was computed from different sets of years for different MOD-LSP parameter sets. For the MOD_IGBP.mode parameter set, the gap-filled 8-day timeseries of MODIS phenology spanning 2000–2016 were aggregated to a single year of climatological mean monthly values. For the NLCD_INEGI parameter sets, the annual cycle was derived in two alternate ways: (1) taken from the single year corresponding to the land cover map, using data from all pixels; or (2) computed as the climatological mean annual cycle over 2000–2016, using only those 30-m land cover pixels that had a stable land cover class between the 2001 and 2011 maps, thereby providing a climatological cycle free of the impacts of land cover change (in which case *lc_year* in the filename was prepended with “s”). In all cases, the string *phen_years* in the filename indicates the years from which the annual cycle was derived.

Thus, the difference between simulations using 2011 and 2001 parameter sets would show the full impact of land cover change and within-class interannual variability in phenology between the years 2001 and 2011, while the difference between simulations using s2011 and s2001 would show the impact of land cover change alone. To assess the impact of interannual variability of phenology alone, an additional set of 17-year monthly time series was prepared for all MOD-LSP parameter sets via computing monthly mean values from the gap-filled 8-day records of LAI, *f*_*canopy*_, and albedo. For the NLCD_INEGI.2001 and NLCD_INEGI.2011 parameter sets, these time series include the impact of land cover change on phenology; however the classification of the pixels in these cases will be that of either year 2001 or 2011.

#### Non-MODIS surface properties

All parameter sets obtained grid cell elevations from the USGS GTOPO30 digital elevation model^6^. For the MOD-LSP parameter sets, GTOPO30 was aggregated to 0.0625° resolution, but L2015 was sub-sampled from earlier 0.125° (12 km) spatial resolution datasets; thus their land masks differ along the coastlines. All parameter datasets used the soil properties of L2015, which were obtained from the FAO-UNESCO Digital Soil Map of the World^35^. Similarly, the MOD-LSP datasets used the conceptual soil parameter values (e.g., *b*_*infilt*_, *Dsmax*, and soil layer thicknesses) of L2015, which were derived via calibration in previous studies^11,14^.

Time- and space-invariant surface properties for each land cover class (e.g., presence of overstory, stomatal and canopy architectural resistance parameters) were taken from the L2015 dataset, which in turn obtained these values from literature^33^. Because the new parameter datasets used different land cover classification schemes than the L2015 dataset, these time- and space-invariant parameters were mapped to classes of MOD_IGBP and NLCD_INEGI from the most appropriate class of the UMD-NLDAS classification as shown in Supplementary Tables 1 and 2, respectively. For the unvegetated open water, perennial ice and snow, and barren soil classes, which were not present in UMD-NLDAS, grassland was chosen as the source class. The impact of this assignment was minimal because the values of LAI, *f*_*canopy*_, and albedo observed by MODIS for these classes corresponded to those of extremely sparse vegetation, essentially rendering the other vegetation parameters (e.g., stomatal and canopy structural resistances) inactive in the VIC model.

## Data Records

All parameter sets discussed herein are available for download at Zenodo^36^. The parameter sets are stored in NetCDF files, formatted for input to the VIC model, version 5.0 and later^10^, in image mode. There are three types of files included in the MOD-LSP project (Table 1): (1) VIC parameter files, which contain grids of land cover class area fractions (“Cv”), spatially-varying annual cycles of monthly phenology variables (“LAI”, “fcanopy”, “albedo”) for all classes, soil properties, and vegetation structural properties that are invariant in time and space; (2) “veg_hist” compressed tar files, each containing 17 yearly files of monthly time-varying phenology variables; and (3) a template (“global_param.template”) for the global parameter file, which specifies the names, locations, and contents of all input and output files, and simulation parameters such as model time step, start and end dates, and model physics options (Supplementary Table 3). Detailed information on parameter file variables and formatting can be found in the VIC model code archive on GitHub (https://github.com/UW-Hydro/VIC).

## Technical Validation

### Evaluation of MOD-LSP phenology

Because MOD-LSP LAI and albedo were only spatially aggregated from their source datasets, which have been extensively validated elsewhere^3,5,16,34,37,38^, a comprehensive validation of those values against observations has not been performed. However, we have evaluated natural *f*_*canopy*_ over much of the United States; and urban LAI and *f*_*canopy*_ over Santa Barbara, CA, USA.

**Fig. 3:**
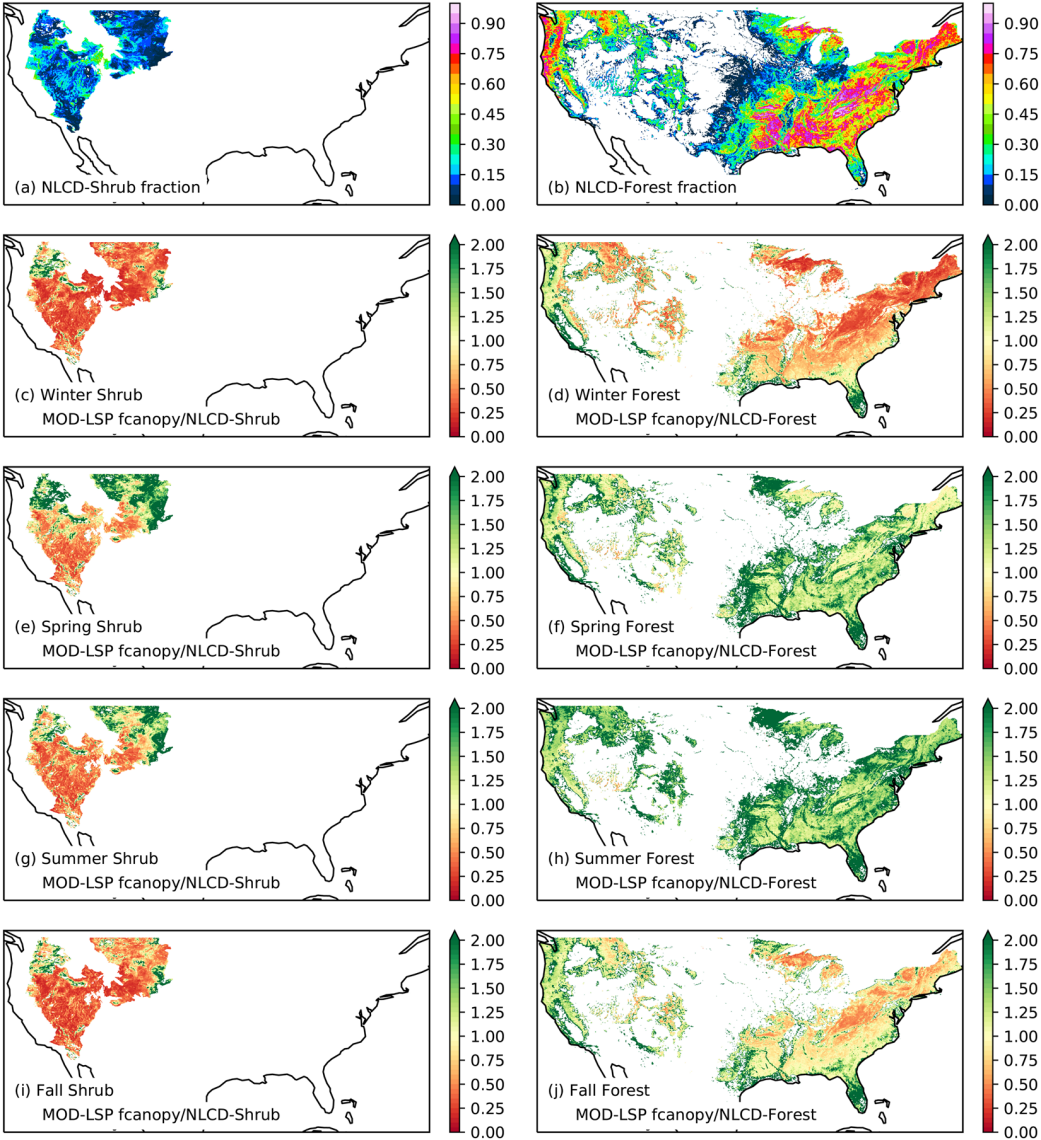
Comparison of the climatological mean seasonal *f*_*canopy*_ over the period 2000–2016 from NLCD_INEGI.2011.2000_2016 parameters to NLCD canopy cover products at 0.0625° (6 km) resolution. (**a**) Shrub fraction from NLCD-Shrub. (**b**) Canopy fraction from NCLD-Forest. (**c**) Ratio of mean winter (January-March) MOD-LSP *f*_*canopy*_ from shrubland and grassland classes to NLCD-Shrub fractions. (**d**) Ratio of mean winter MOD-LSP *f*_*canopy*_ from all forest classes to NLCD-Forest canopy fractions. (**e**–**j**) Same as (**c**,**d**) but for spring (April-June), summer (July-September), and fall (October-December). White pixels indicate no data available.

To evaluate the values of *f*_*canopy*_, two independent datasets were used: the NLCD US Forest Service Percent Tree Canopy Cartographic product^36^ (NLCD-Forest hereafter) and the NLCD Shrubland product^37^ (NLCD-Shrub hereafter). These datasets were aggregated from 30-m to 0.0625° resolution over the NLCD_INEGI.2011 land cover classification in a similar fashion to the MODIS observations. The MOD-LSP estimates of *f*_*canopy*_ substantially outperformed L2015 in reproducing the statistics of the NLCD-Shrub and NLCD-Forest products (Table 4). Across the entire domain, mean annual *f*_*canopy*_ from NLCD_INEGI.s2011 over the period 2000–2016 underestimated the NLCD-Shrub area fractions and overestimated NLCD-forest canopy fractions by factors of 0.70 and 1.24, respectively, yielding RMSE values of 0.09 and 0.21, respectively. In contrast, the L2015 parameter set, which implicitly assumed *f*_*canopy*_ = 1.0 in all vegetated classes, grossly overestimated the NLCD-Shrub and NLCD-Forest values by factors of 7.24 and 2.28, and yielded RMSE values of 0.87 and 0.61. At the seasonal scale (Table 4, Fig. 3 and Supplementary Fig. 2), *f*_*canopy*_ exhibited a strong seasonal cycle, with a minimum in winter and a maximum in spring or summer. Over shrublands and grasslands, *f*_*canopy*_ both underestimated and showed less spatial variation than the NLCD-Shrub values in all seasons, leading to worse underestimation where NLCD-Shrub coverage values were higher, e.g., at higher elevations (Fig. 3). Over forests, *f*_*canopy*_ underestimated NLCD-Forest values in winter, overestimated them in summer, and was relatively less biased in spring and fall, particularly in the eastern United States (Fig. 3). The seasonality is due to both to real phenology (leaf growth and loss in deciduous vegetation) and to biases inherent in Eq. (2) (variation of the solar angle and canopy shading)^39^. Nevertheless, a prior study^24^ found that VIC simulations using *f*_*canopy*_ values derived from MODIS NDVI via Eq. (2) resulted in substantial improvements over using *f*_*canopy*_ = 1.0 in simulated ET at 60+ shrubland and forest AMERIFLUX^40^ eddy covariance tower sites.

A comprehensive evaluation of urban LAI and *f*_*canopy*_ from MOD-LSP has not been undertaken. However, over Santa Barbara, CA, July values of LAI and *f*_*canopy*_ compare favorably to 71 randomly-sampled field observations from a prior study^41^. Observed LAI values had a mean of 1.0035 and standard error of 0.1097. The NLCD_INEGI.s2011 LAI values, averaged over all urban classes in the grid cells containing Santa Barbara, had a mean of 0.8843. The study reported canopy coverage values of 0.20 (from the UFORE model) and 0.254 (estimated from high-resolution imagery by the city of Santa Barbara). The NLCD_INEGI.s2011 July urban *f*_*canopy*_ values had a mean of 0.2256.

Because the MOD-LSP LAI timeseries changed source datasets (from MOD15A2H.006, Terra sensor only; to MCD15A2H.006, Terra + Aqua sensors) after the first 1.5 years of record, changes in statistics are a potential issue. Prior studies^37^ have found that the two products yielded generally similar values, particularly over forests and grasslands, but exhibited different temporal mean and variance over highly variable biomes such as croplands. However, we expect the impact on the climatological mean values to be small, as the time span derived from the MOD15A2H.006 product comprised less than 10% of the record.

### Comparison of land cover distributions

**Fig. 4:**
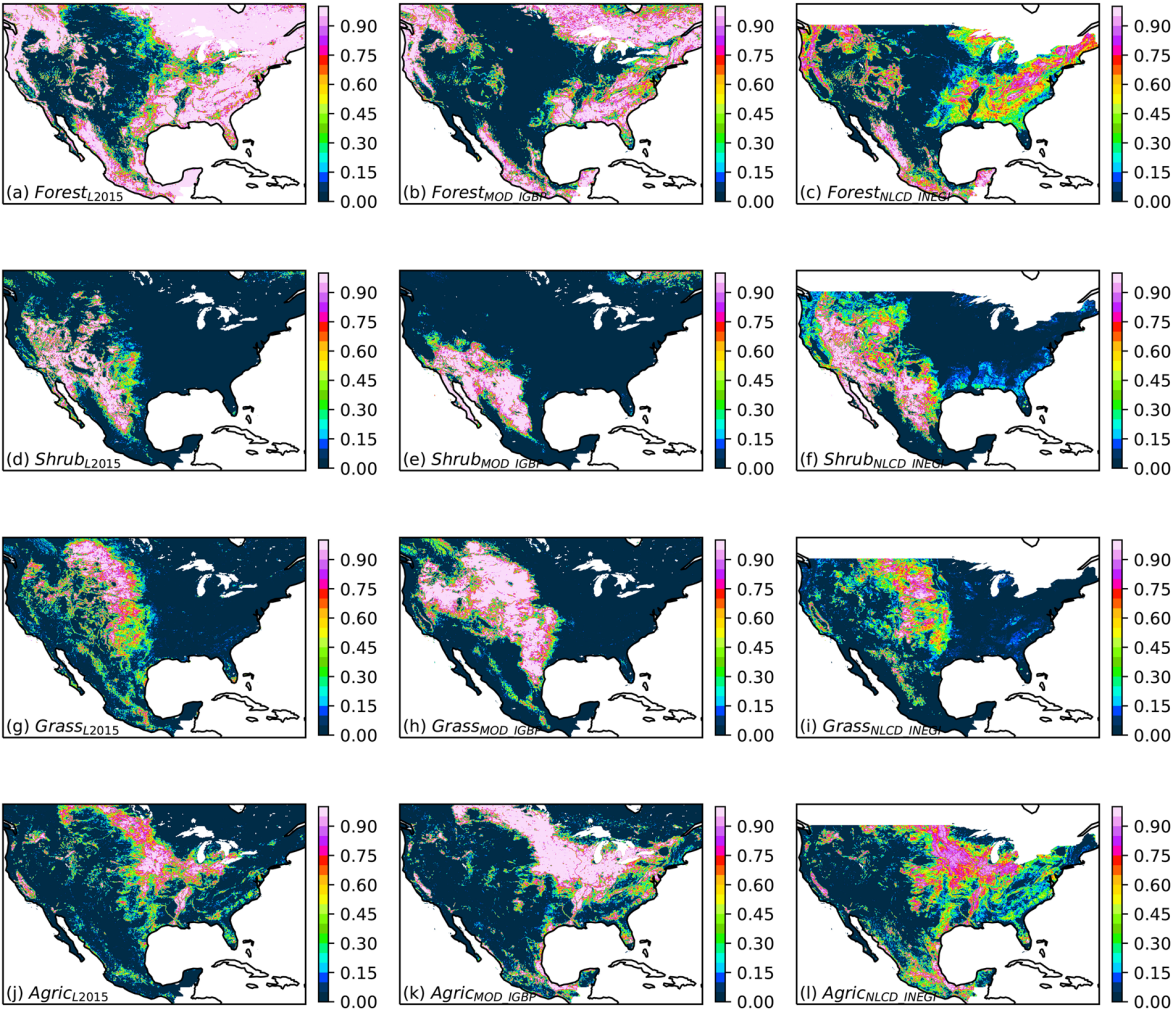
Comparison of geographic distributions of land cover types in L2015, MOD_IGBP, and NLCD_INEGI.2011. Maps show the total area coverage fraction of (**a**–**c**) all forest classes; (**d**–**f**) all shrub classes; (**g**–**i**) all grassland classes; (**j**–**l**) all agricultural and pastoral classes.

The L2015, MOD_IGBP, and NLCD_INEGI.2011 land cover classifications generally agree on the geographic distributions of major land cover categories, although there are notable differences (Fig. 4). For the “forest” category, all three land cover datasets show high coverage in eastern Canada and northeastern US, the southeastern US, southern Mexico, mountains west of 100° W, and the northern Pacific coast (Fig. 4a–c). However, L2015 has higher forest coverage around the fringes of these regions than MOD_IGBP, while NLCD_INEGI has lower coverage. This might be due in part to the spatial resolutions of the underlying land cover products (1 km for UMD, 500 m for MOD12Q1.005, and 30 m for NLCD and INEGI). In particular, NLCD does not include classes for mixes of trees and grass. For shrublands (Fig. 4d–f) and grasslands (Fig. 4g–i), the classifications agree on the overall locations of these classes but differ on the boundary between shrubland and grassland. L2015 is more similar to NLCD_INEGI in its differentiation between shrublands and grasslands, with shrublands extending into the northwestern US, while in MOD12Q1.005 shrublands are confined to the southwestern US and western Mexico. The products agree on agricultural areas (Fig. 4j–l), but MOD_IGBP has higher coverage in most of the US and Canada, while NLCD_INEGI exhibits greater extent in Texas and Mexico.

### Comparison of phenology distributions

The improvements of MOD-LSP phenology relative to L2015 are evident when comparing distributions over North America. For the “mixed forest” class (Supplementary Fig. 3), the July LAI of L2015 contains regions of homogeneous values with abrupt boundaries, while MOD_IGBP.mode exhibits a physically reasonable distribution. The L2015 July *f*_*canopy*_ is 1.0 everywhere, while MOD_IGBP.mode varies from 0.5 to 1.0 and shows a similar spatial distribution to LAI. Similarly, the L2015 July albedo is 0.18 everywhere, while that of MOD_IGBP.mode ranges from 0.10 to 0.18. Similar problems are evident in the L2015 cropland class, relative to that of MOD_IGBP.mode (Supplementary Fig. 4).

**Fig. 5:**
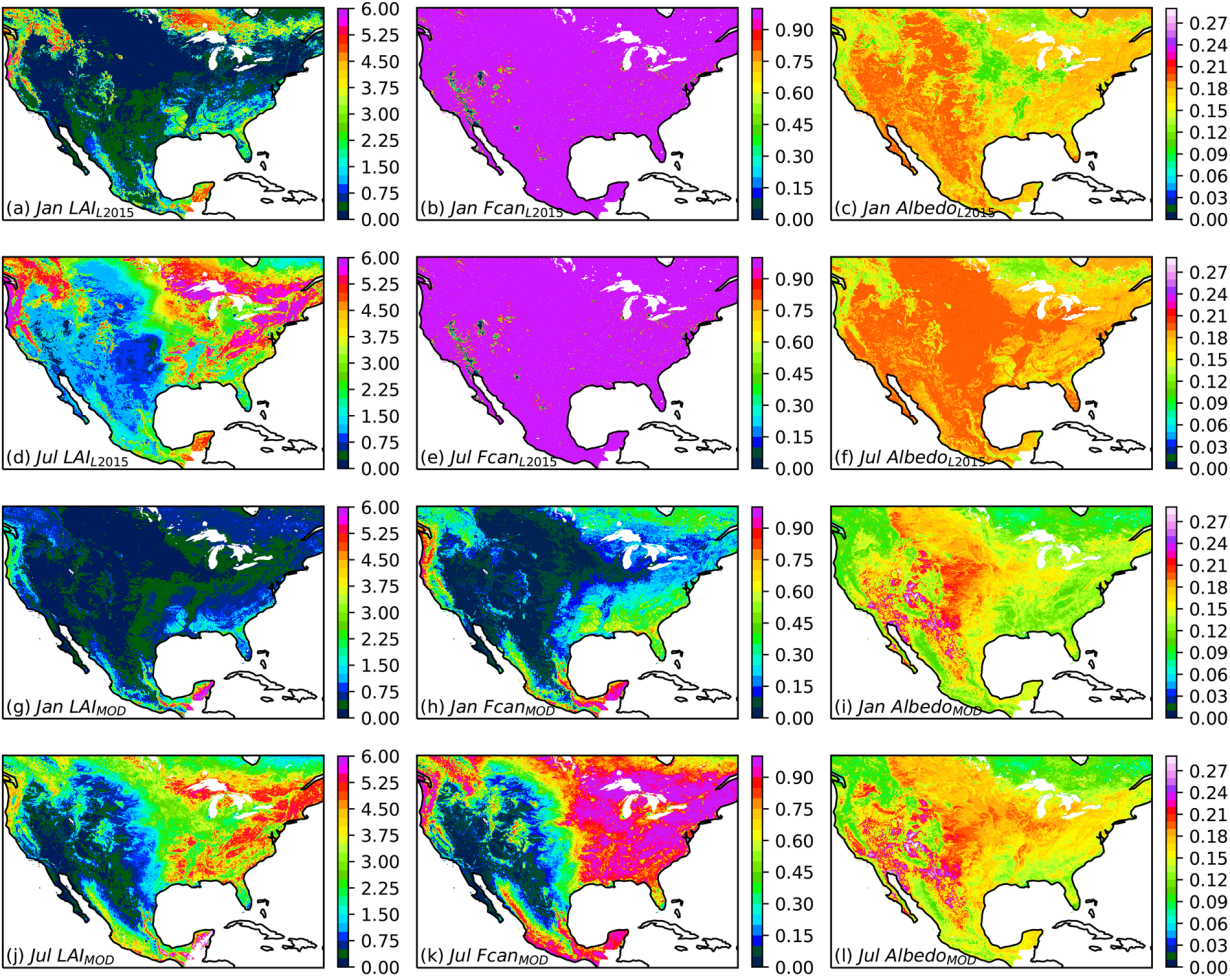
Comparison of climatological average January and July phenology of L2015 and MOD_IGBP datasets for the area-weighted average across all land cover classes. (**a**–**f**) LAI, *f*_*canopy*_, and albedo from L2015, for (**a**–**c**) January and (**d**–**f**) July; (**g**–**l**) LAI, *f*_*canopy*_, and albedo from MOD_IGBP, for (**g**–**i**) January and (**j**–**l**) July.

MOD-LSP and L2015 parameter sets also differ in total “scene” phenology, or the area-weighted average phenology across all land cover classes (Fig. 5). In January, the “scene” LAI of L2015 (Fig. 5a) is substantially higher than that of MOD_IGBP.mode (Fig. 5g), particularly in the northern forests. This could be due to L2015 holding LAI constant year-round for evergreen forest and possible residual impacts from clouds and snow in the MOD-LSP processing. In July, however, the LAI of L2015 (Fig. 5d) and MOD_IGBP.mode (Fig. 5j) look more similar. *f*_*canopy*_ remains 1.0 year-round in vegetated cells in L2015 (Fig. 5b,e) but displays a seasonal cycle in MOD_IGBP.mode (Fig. 5h,k). For albedo, spatial variations are evident in L2015, ranging from 0.12 to 0.20, but there are no temporal variations except over crops (Fig. 5c,f). The MOD_IGBP.mode albedo exhibits much larger spatial variability than L2015, ranging from 0.10 to 0.30 (Fig. 5i,l).

### Impacts on hydrology

**Fig. 6:**
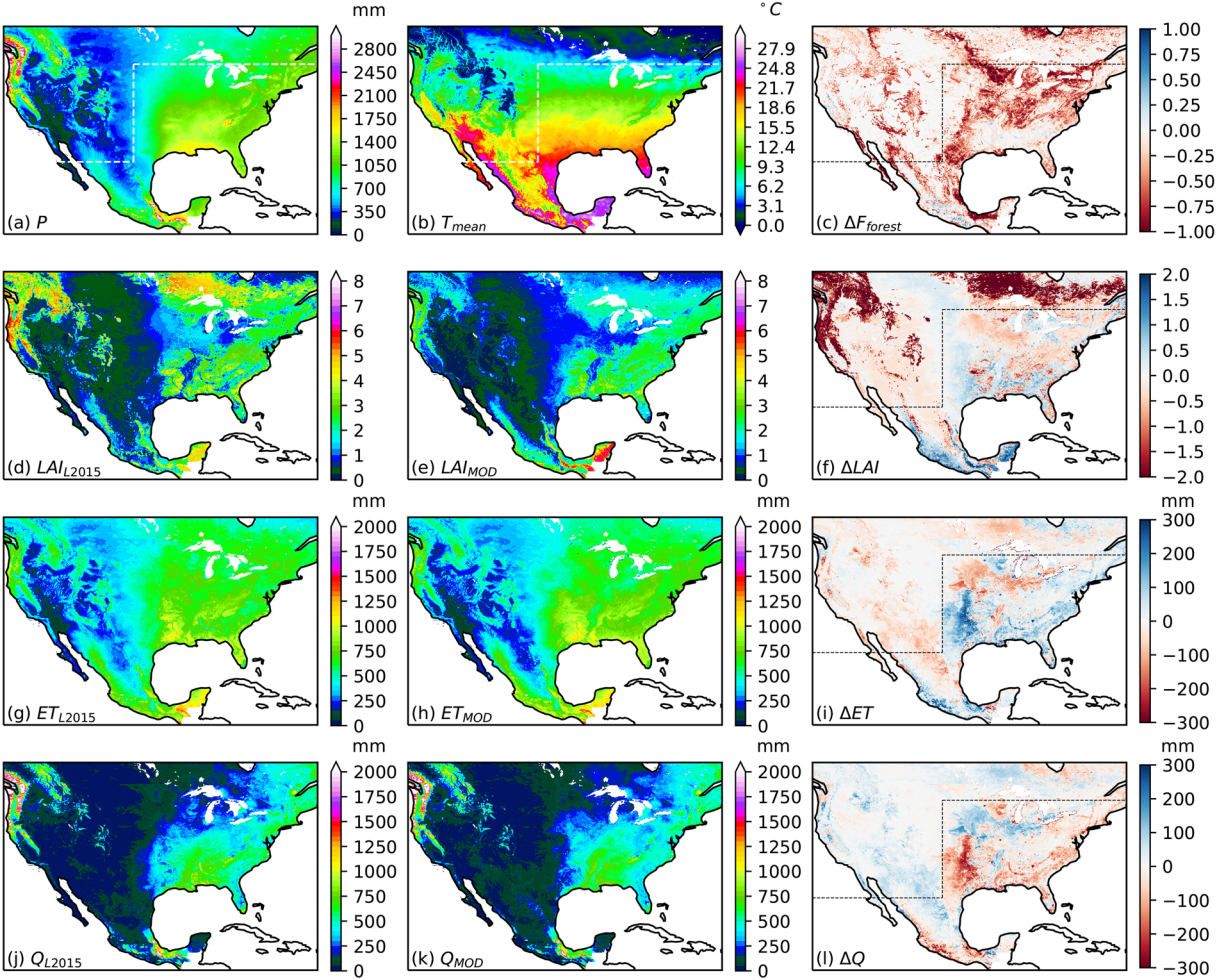
Comparison of annual hydrologic terms between simulations using the L2015 and MOD_IGBP datasets over 1981–2013 (for *f*_*canopy*_ uniformly held at 1.0). (**a**,**b**) Mean annual precipitation (P) and temperature (T) from L2015; (**c**) difference in forest area fraction between L2015 and MOD_IGBP; (**d**–**f**) mean annual LAI from L2015 and MOD_IGBP simulations and their difference; (**g**–**i**) mean annual ET from L2015 and MOD_IGBP simulations and their difference; (**j**–**l**) mean annual Q from L2015 and MOD_IGBP simulations and their difference. Dashed lines denote boundary between cold or dry climates and warm or wet climates.

Differences in land cover distributions and phenology between L2015 and MOD-LSP impacted water and energy fluxes obtained from the VIC simulations. Holding *f*_*canopy*_ constant at 1.0 (Fig. 6), differences in mean annual evapotranspiration (ET) and runoff (Q) between simulations using the MOD_IGBP and L2015 parameter sets were positively correlated in the case of ET and negatively correlated in the case of Q with the difference in mean annual LAI between the two parameter sets. In the warmer or wetter regions of the domain (south and east of the dashed lines), ET and Q exhibit sensitivities to differences in LAI of about +/−200 mm y^−1^ per unit change in LAI. However, the differences diminish in the cooler or drier portions of the domain (to the north and west of the dashed lines).

Similarly, allowing *f*_*canopy*_ to vary resulted in changes of about −/+50 mm y^−1^ per unit change in *f*_*canopy*_ in the warmer and wetter portions of the domain, due to reduced canopy evaporation being outweighed by the increased soil evaporation and transpiration from increased throughfall (Supplementary Fig. 5). Sensitivities to changes in *f*_*canopy*_ diminished as climate became drier, but where annual precipitation (P) fell primarily as snow, the sensitivities of ET and Q to changes in *f*_*canopy*_ changed sign, due to reduced snow storage in the forest canopy, where aerodynamic resistance is lower than at the surface of the ground snow pack. The total difference between MOD_IGBP with varying *f*_*canopy*_ and L2015 was dominated by the difference due to LAI, but *f*_*canopy*_ differences reinforced the LAI impacts in snowy mountains.

The NLCD_INEGI products show substantial land cover change between 2001 and 2011 (Supplementary Fig. 6). Forests experienced substantial losses in the southeastern US, the northern Pacific coast, and much of Mexico. Shrublands and grasslands expanded in the southeastern and northwestern US, but shrank in northern Mexico. Agriculture expanded substantially in Mexico and cities expanded everywhere. At smaller scales (boxes 1–4), the NLCD_INEGI products show the loss of forest to shrub/grassland in response to drought and fire in the Rocky Mountains^42^ (box 1); a mix of forest-shrub, crop-forest, and crop-urban conversion around Lake Michigan (box 2); forest-shrub, forest-crop, and shrub-crop conversion in northwestern Mexico due to pasture clearing and agricultural expansion^43^ (box 3); and forest-shrub and forest-urban conversion around Atlanta (box 4).

**Fig. 7:**
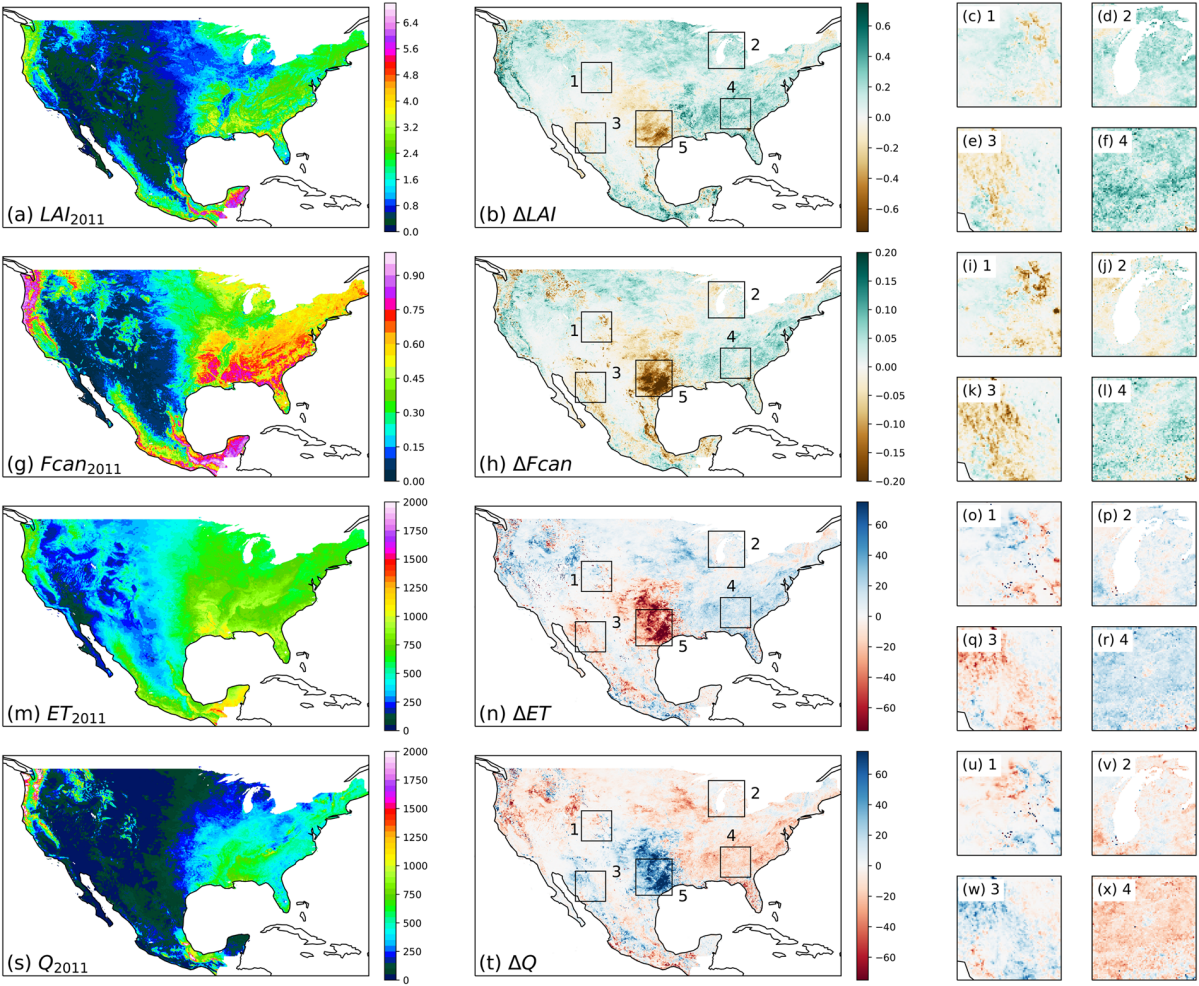
Changes in the phenology and annual hydrologic fluxes between 2001 and 2011 in the NLCD_INEGI.2001.2001_2001 and NLCD_INEGI.2011.2011_2011 parameter sets. (**a**) Mean annual LAI in 2011; (**b**) change in mean annual LAI between 2001 and 2011; (**c**–**f**) magnification of boxes 1–4 in (**b**). (**g**–**l**) Same as (**a**–**f**) for mean annual *f*_*canopy*_. (**m**–**r**) Same as (**a**–**f**) for mean annual ET. (**s**–**x**) Same as (**a**–**f**) for mean annual Q.

These changes in land cover, coupled with interannual variations in phenology (via the NLCD_INEGI.2001.2001_2001 and NLCD_INEGI.2011.2011_2011 parameter sets), led to changes in hydrologic fluxes (Fig. 7). Forest-shrub conversion in the Rocky Mountains (box 1) and forest-agriculture conversion in northwestern Mexico (box 3) were accompanied by reductions in LAI and *f*_*canopy*_, leading to reduced ET and increased Q. However, crop-urban conversion around Lake Michigan (box 2) and forest-urban conversion around Atlanta (box 4) did not lead to reductions in LAI or *f*_*canopy*_ and had minimal impacts on hydrology. Furthermore, interannual fluctuations in LAI and *f*_*canopy*_ in eastern Texas (box 5) led to substantial changes in hydrology without being accompanied by substantial land cover change.

## Usage Notes

To run VIC with the parameter files described herein requires corresponding domain files, available on Zenodo^44^. Instructions for configuring VIC to use these parameter files are available in the MOD-LSP User Guide included with the parameter files^36^.

## Supplementary Information

### ISA-Tab metadata file

Download metadata file

### Supplementary information

Supplementary Material

## Data Availability

The MOD-LSP parameter sets were created via Python scripts (using the xarray package)^45^ archived on GitHub^46^ (https://github.com/tbohn/VIC_Landcover_MODIS_NLCD_INEGI/releases/tag/v1.6). The NLCD_INEGI harmonized US-Mexico land cover classifications are available for download at Zenodo^47^, with scripts archived on GitHub^30^ (https://github.com/tbohn/NLCD_INEGI/releases/tag/v1.6). The L2015 parameter set was converted from ascii to VIC-5-compliant NetCDF format by the “tonic” tool (https://github.com/UW-Hydro/tonic/releases/0.2).
