# Supplementary material for: MOD-LSP, MODIS-based parameters for hydrologic modeling of North American land cover change
Source: Sci Data. 2019 Aug 9;6:144. doi: 10.1038/s41597-019-0150-2 (PMC6689012; doi:10.1038/s41597-019-0150-2)
Supplement: Supplementary file 2 — Supplementary Material [file 41597_2019_150_MOESM2_ESM.pdf]

Supplemental materials for: MOD-LSP, MODIS-based parameters for hydrologic modeling of North American land cover change

Theodore J. Bohn[1] and Enrique R. Vivoni[1,2]

1. School of Earth and Space Exploration,
Arizona State University, Tempe, AZ 85287

2. School of Sustainable Engineering and the Built Environment,
Arizona State University, Tempe, AZ 85287

Submitted to *Scientific Data*

April 7, 2019

Table of Contents

Tables 3

Supplementary Table 1. 3

Supplementary Table 2. 4

Supplementary Table 3. 5

Figures 6

Supplementary Figure 1. 6

Supplementary Figure 2. 8

Supplementary Figure 3. 9

Supplementary Figure 4. 10

Supplementary Figure 5. 11

Supplementary Figure 6. 12

Tables

Supplementary Table 1. Mapping between MOD-IGBP classes and UMD-NLDAS classes from which structural parameters were taken.

MOD-IGBP		UMD-NLDAS	
**Code**	**Names**	**Code**	**Name**
0	Open Water	10	Grassland
1	Evergreen Needleleaf Forest	1	Evergreen Needleleaf Forest
2	Evergreen Broadleaf Forest	3	Evergreen Broadleaf Forest
3	Deciduous Needleleaf Forest	2	Deciduous Needleleaf Forest
4	Deciduous Broadleaf Forest	4	Deciduous Broadleaf Forest
5	Mixed Forest	5	Mixed Forest
6	Closed Shrubland	8	Closed Shrubland
7	Open Shrubland	9	Open Shrubland
8	Woody Savanna	6	Woodland
9	Savanna	7	Savanna
10	Grassland	10	Grassland
11	Wetland	10	Grassland
12	Cropland	11	Cropland
13	Urban	10	Grassland
14	Crop-Natural Mosaic	11	Cropland
15	Perennial Ice/Snow	10	Grassland
16	Barren	10	Grassland

Supplementary Table 2. Mapping between NLCD-INEGI classes and UMD-NLDAS classes from which structural parameters were taken.

NLCD-INEGI		UMD-NLDAS	
**Code**	**Names**	**Code**	**Name**
11	Open Water	10	Grassland
12	Perennial Ice/Snow	10	Grassland
21	Developed, Open Space	10	Grassland
22	Developed, Low Intensity	10	Grassland
23	Developed, Medium Intensity	10	Grassland
24	Developed, High Intensity	10	Grassland
31	Barren Land	10	Grassland
41	Deciduous Forest[1]	4	Deciduous Broadleaf Forest
42	Evergreen Forest[1]	1	Evergreen Needleleaf Forest
43	Mixed Forest	5	Mixed Forest
52	Shrub/Scrub	9	Open Shrubland
71	Grassland/Herbaceous	10	Grassland
81	Pasture and Hay	11	Cropland
82	Cultivated Crops	11	Cropland
90	Woody Wetlands	10	Grassland
95	Emergent Herbaceous Wetlands	10	Grassland

[1]The NLCD classes of "Deciduous Forest" and "Evergreen Forest" (codes 41 and 42) were interpreted to correspond to broadleaf and needleleaf forest, respectively, in Mexico. Over the CONUS_MX domain, Deciduous Needleleaf Forest is extremely rare, so that the NLCD Evergreen Forest class essentially denotes Needleleaf Forest. Similarly, in the UMD-NLDAS dataset, the time-invariant structural parameters for Deciduous Broadleaf Forest were the same as those for Evergreen Broadleaf Forest, so that our interpretation did not adversely impact parameter values.

Supplementary Table 3. Explanation of placeholders in global_param.template file.

Option	Description	Default
<DOMAIN_FILE>	Path/name of the domain file. The land mask in the domain file determines the spatial domain of the simulation. The spatial dimensions (resolution, numbers of rows and columns, latitudes and longitudes of grid cell centers) must match those in the desired VIC parameter file exactly. Domain files covering the CONUS_MX and USMX domains are available on Zenodo at (https://zenodo.org/record/2564019).	None.
<PARAM_FILE>	Path/name of the desired parameter file.	None.
<LAI_SRC>,<br><FCAN_SRC>,<br><ALB_SRC>	Options to tell the VIC model where to obtain phenology variables from. To use the repeating annual cycles of monthly values stored in the parameter file, these should be set to FROM_VEGPARAM. To use the monthly time series of phenology from the veg_hist files, these should be set to FROM_VEGHIST (see below for instructions on preparing the veg_hist files). Because the L2015 parameter file does not contain f_{canopy} values and has no associated veg_hist file, <FCAN_SRC> must be set to FROM_DEFAULT when using the L2015 parameter file (which instructs VIC to use a value of 1.0 everywhere).	<FCAN_SRC>:<br>FROM_DEFAULT<br>All others:<br>FROM_VEGPARAM
<FORCING_DIR>	Path of the directory containing meteorological forcing files.	None.
<FORCING_PFX>	Prefix of meteorological forcing files. Forcing files are assumed to be named as *forcing_pfx*.YYYY.nc, where YYYY = the year covered by the file.	None.
<VEGHIST_DIR>	Path of the directory containing veg_hist files.	Use repeating cycle from parameter file.
<VEGHIST_PFX>	Prefix of veg_hist files. Veg_hist files are assumed to be named as *veghist_pfx*.YYYY.nc, where YYYY = the year covered by the file.	Use repeating cycle from parameter file.

Figures

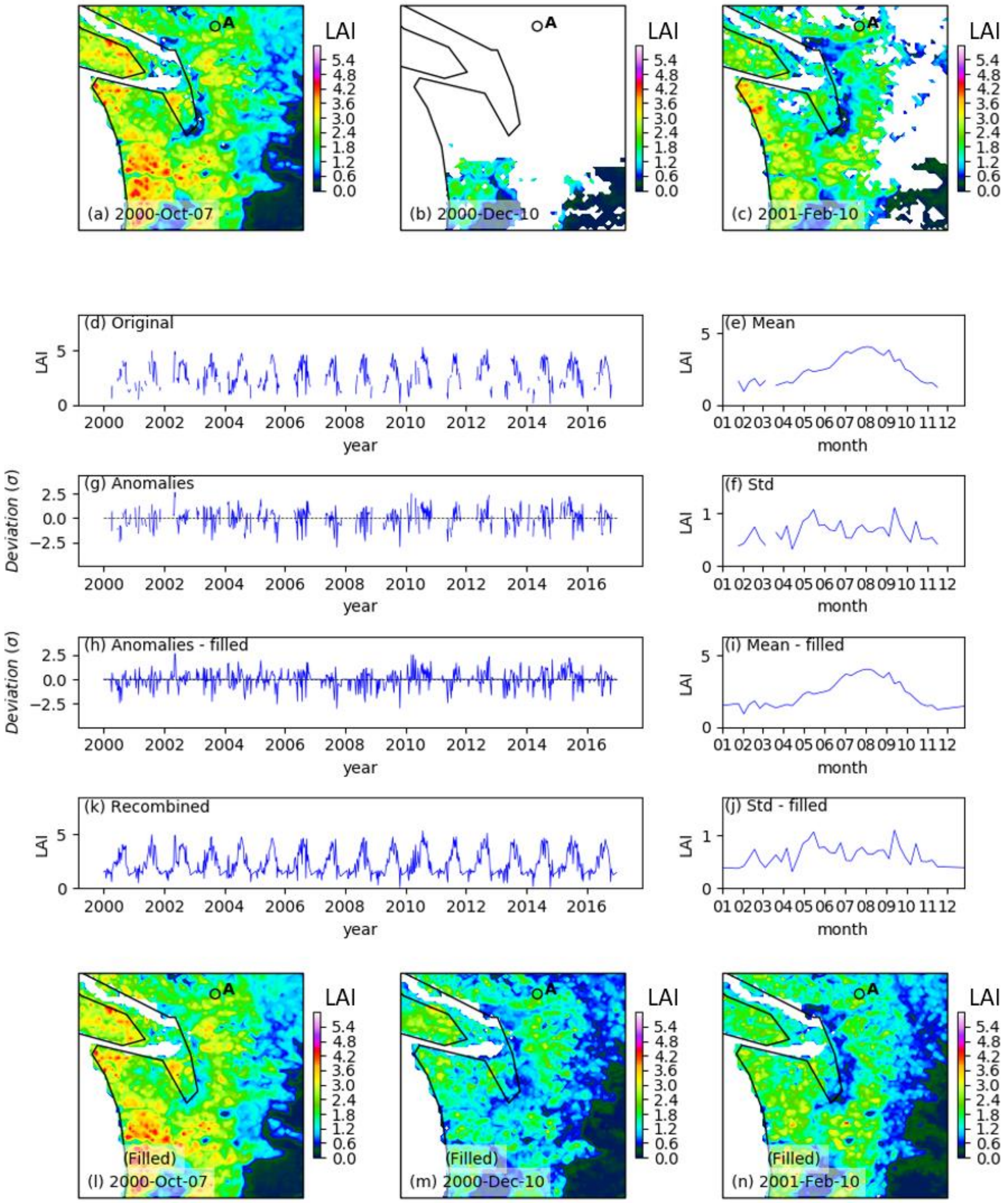

Supplementary Figure 1. Demonstration of gap filling process using LAI over the geographic region bounded by 45-50° N latitude and 120-125° W longitude, centered on the Puget Sound region of western WA, USA. (a-c) Maps of 8-day MOD15A2H.006 LAI aggregated over land cover classes from the MOD12Q1.051 IGBP land cover classification at 0.0625° resolution, for

days 2000-Oct-07, 2000-Dec-10, and 2001-Feb-10. Gaps (white pixels) are present due to low solar angle, snow, and clouds. (d) Time series of LAI (from MOD and MCD products) for mixed forest, from the grid cell denoted by "A" in panels (a-c). (e-f) Climatological mean and standard deviation of LAI for each 8-day interval over the period 2000-2016. (g) Anomalies of LAI relative to the climatological mean, in units of numbers of standard deviations from the mean. (h-j) Climatological mean, standard deviation, and anomalies after gap filling. (k) Gap-filled time series created by recombining gap-filled mean, standard deviation, and anomalies of panels (h-j). (l-n) Gap-filled versions of the maps in panels (a-c).

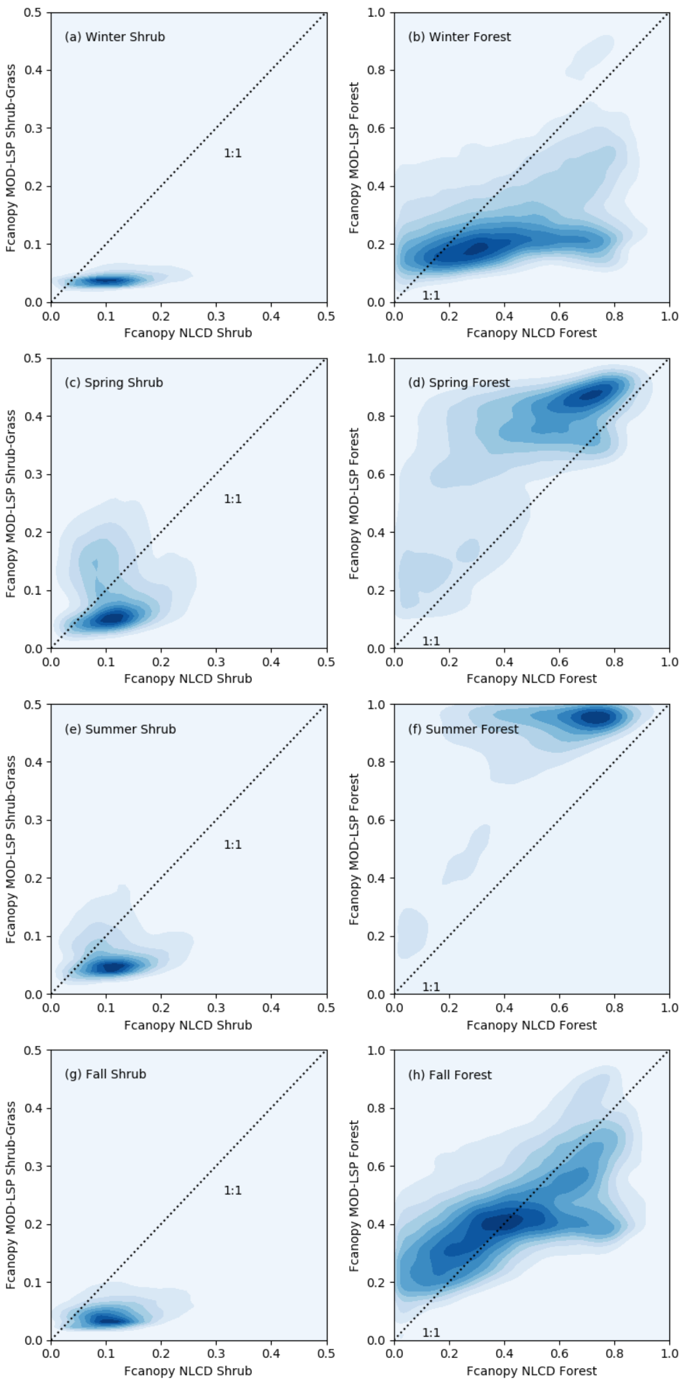

Supplementary Figure 2. Comparison of the climatological mean seasonal f_{canopy} over the period 2000-2016 from NLCD_INEGI.2011.2000_2016 parameters to NLCD canopy cover products at 0.0625° (6 km) resolution. (a) Mean winter (January-March) MOD-LSP f_{canopy} from shrubland and grassland classes vs. NLCD-Shrub fractions. (b) Mean winter MOD-LSP f_{canopy} from all forest classes vs. NLCD-Forest canopy fractions. (c-d), (e-f), and (g-h) Same as (a-b) but for spring (April-June), summer (July-September), and fall (October-December).

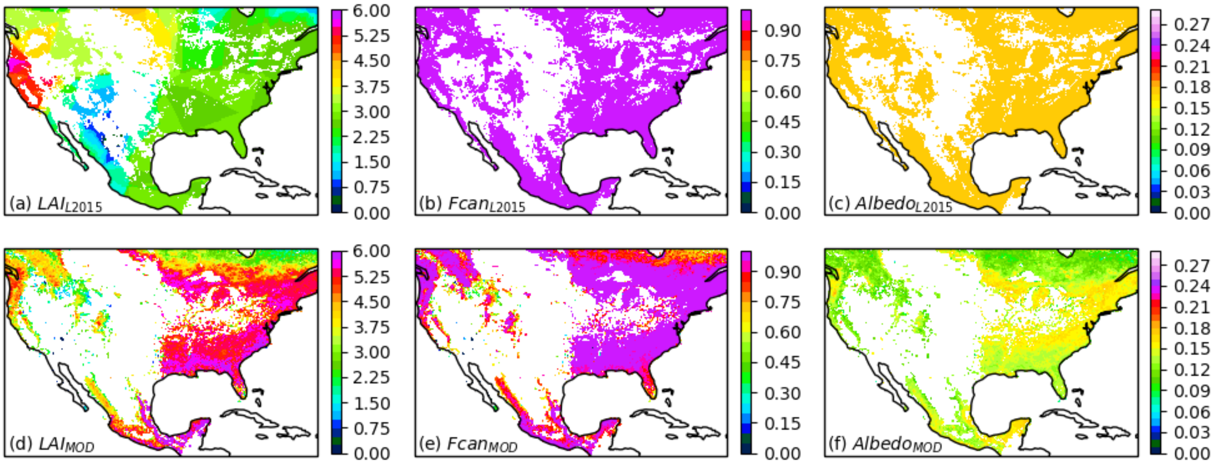

Supplementary Figure 3. Comparison of climatological average July phenology between L2015 and MOD-LSP MOD_IGBP datasets for the mixed forest class. (a-c) LAI, f_{canopy}, and albedo from L2015; (d-f) LAI, f_{canopy}, and albedo from MOD-LSP MOD_IGBP. White pixels indicate class not present.

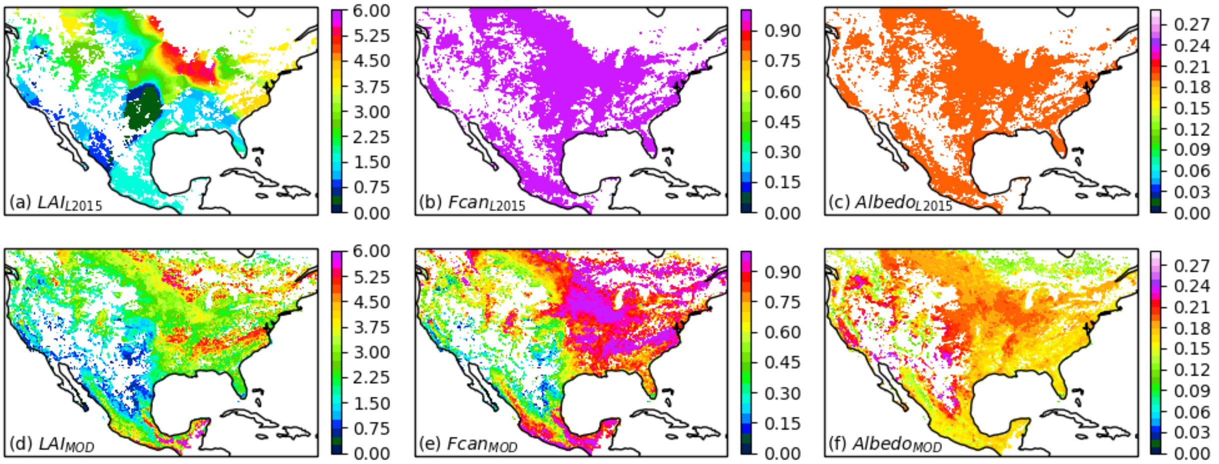

Supplementary Figure 4. Comparison of climatological average July phenology between L2015 and MOD-LSP MOD_IGBP datasets for the cropland class. (a-c) LAI, f_{canopy}, and albedo from L2015; (d-f) LAI, f_{canopy}, and albedo from MOD-LSP MOD_IGBP. White pixels indicate class not present.

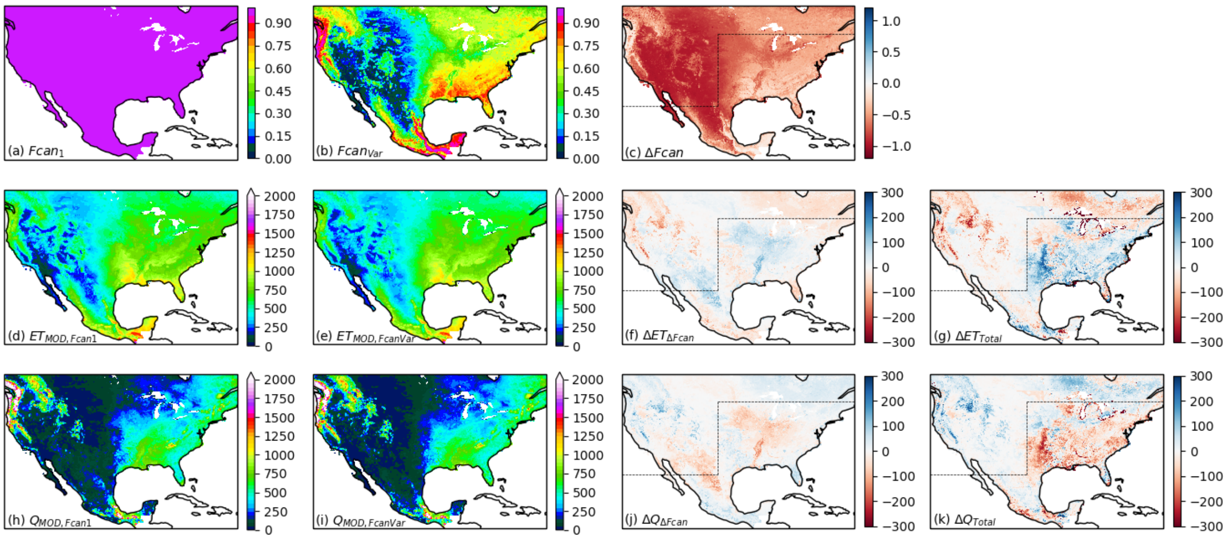

Supplementary Figure 5. Comparison of annual hydrologic terms between simulations with uniform and spatially-varying f_{canopy}, using the MOD_IGBP dataset over the period 1981-2013. (a-c) Uniform f_{canopy}, mean annual spatially-varying f_{canopy}, and their difference; (d-f) mean annual ET from uniform and spatially-varying f_{canopy} simulations and their difference; (h-j) mean annual Q from uniform and spatially-varying f_{canopy} simulations and their difference; (g) and (k) total difference in (g) ET and (k) Q between MOD-IGBP spatially-varying f_{canopy} and L2015 uniform f_{canopy} simulations. Dashed lines denote boundary between cold/dry climates and warm/wet climates.

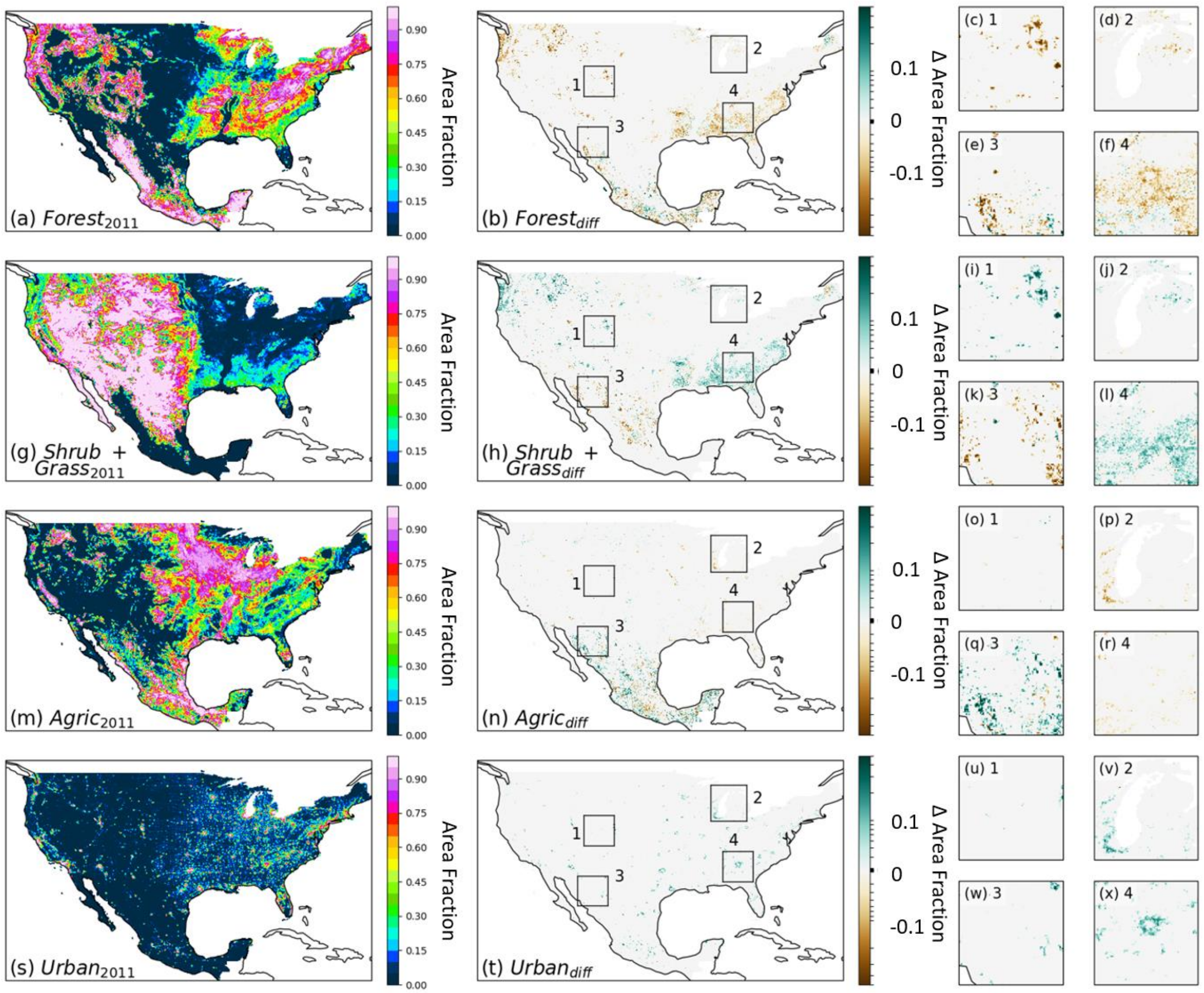

Supplementary Figure 6. Changes in the geographic distributions of broad land cover classes between 2001 and 2011 in the NLCD_INEGI parameter sets. (a) Distribution of all forest classes in 2011; (b) change in distribution of forest between 2001 and 2011; (c-f) magnification of boxes 1-4 in (b). (g-l) same as (a-f) for combined areas of shrubland and grassland classes. (m-r) same as (a-f) for combined areas of agricultural and pastoral classes. (s-x) same as (a-f) for combined areas of urban classes.
